# Cotranslational Protein Folding and Terminus Hydrophobicity

**DOI:** 10.1155/2011/176813

**Published:** 2011-06-06

**Authors:** Sheenal Srivastava, Yumi Patton, David W. Fisher, Graham R. Wood

**Affiliations:** Department of Statistics, Macquarie University, Sydney, NSW 2109, Australia

## Abstract

Peptides fold on a time scale that is much smaller than the time required for synthesis, whence all proteins potentially fold cotranslationally to some degree (followed by additional folding events after release from the ribosome). In this paper, in three different ways, we find that cotranslational folding success is associated with higher hydrophobicity at the N-terminus than at the C-terminus. First, we fold simple HP models on a square lattice and observe that HP sequences that fold better cotranslationally than from a fully extended state exhibit a positive difference (N−C) in terminus hydrophobicity. Second, we examine real proteins using a previously established measure of potential cotranslationality known as ALR (Average Logarithmic Ratio of the extent of previous contacts) and again find a correlation with the difference in terminus hydrophobicity. Finally, we use the cotranslational protein structure prediction program SAINT and again find that such an approach to folding is more successful for proteins with higher N-terminus than C-terminus hydrophobicity. All results indicate that cotranslational folding is promoted in part by a hydrophobic start and a less hydrophobic finish to the sequence.

## 1. Background

An understanding of protein folding is keenly sought for a variety of oft-stated reasons. From a theoretician's perspective, hydrophobic collapse of the string of residues is often conjectured to be a key driver of protein folding [1]. Under such collapse, the manner of folding will be to some extent determined by the hydrophobicity profile. On the other hand, from an experimentalist's perspective, cotranslational folding is acknowledged to occur for certain proteins. Marrying these two perspectives causes us to ask whether there is a hydrophobicity profile that is compatible with and that may even assist in driving cotranslational folding. In this paper, we find evidence in three independent ways that greater hydrophobicity at the N-terminus than at the C-terminus is associated with cotranslational folding. That cotranslational folding occurs (and may be supported by an associated hydrophobicity pattern) underpins this paper, so we now review this process, together with evidence of asymmetry in protein folding from other sources.

Phillips [2] noted over forty years ago, in expounding the structure of the hen egg-white lysozyme molecule, evidence of the nonuniform distribution of hydrophobic residues in a protein and had the bravery to suggest that this may be an indicator of cotranslational folding. The first 40 residues of the N-terminus sequence forms a compact globular core with hydrophobic side chains, while the last 20 residues are folded around this hydrophobic core to give the final lysozyme structure. Thus, in this case, the nitrogen end would appear to fold before the carbon end of the protein. On the other hand, White and Jacobs [3] later found that the distribution of hydrophobic residues in the majority of 5,247 protein chains was random; we comment in the discussion on this last result.

Experimental studies have since shown that there is ample time for a protein to fold while it is still in its nascent state [4]. Cotranslational folding has been solidly evidenced in the Semliki Forest virus capsid protein (one of the five that makes up the Semliki Forest virus polyprotein). It is produced at the amino terminus of the polyprotein and possesses enzymatic activity which allows it to cleave from the remainder before release of the polyprotein from the ribosome [5], so indicating that functionally active structures form well before synthesis is complete. An abundance of experimental evidence for cotranslational folding appears in works such as [6–11].

Cotranslation is an inherently asymmetric process. Such asymmetry in protein folding has been noted in several studies which add computation to the results of raw experimentation. Almost two decades ago, Alexandrov [12] noted that the N-terminus was more compact than the C-terminus in a set of 215 protein structures. More recently, Røgen [13] discovered a difference between the nitrogen and carbon terminals in all then current CATH domains [14] using the Gauss integral, a mathematical construct commonly used in knot theory. Norcross and Yeates [15] were able to infer, from the final fold, most likely paths to that fold and found that folding was more likely to have started towards the nitrogen terminus than the carbon terminus. Recently, Rhodri Saunders et al. (Oxford, private communication) observed asymmetry in the folding of simple HP lattice models. They found, using a different folding algorithm to that used here, that HP lattice sequences reaching their global conformation cotranslationally exhibit a decrease in hydrophobicity throughout their length.

**Figure 1:**
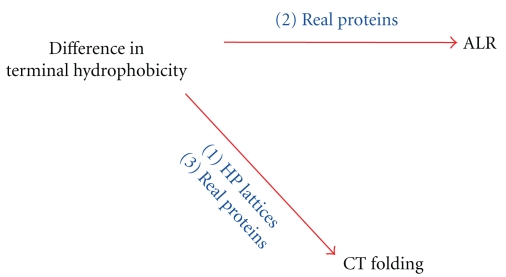
Three results, independently showing that a reduction in hydrophobicity from start to end is associated with cotranslational folding, are summarised. These are (1) a positive correlation between the difference (N−C) in terminus hydrophobicity and cotranslational folding in HP models, (2) a positive correlation between ALR (considered a measure of cotranslational folding in real proteins) and the difference in terminus hydrophobicity, and (3) real proteins that fold more successfully using a cotranslationally based structure prediction algorithm have more hydrophobic N-terminus than C-terminus.

In summary, there is both experimental and computational evidence of asymmetry in protein folding. Here, we investigate, using both HP models and real proteins, whether this is associated with a particular pattern of hydrophobicity along the protein. The evidence is necessarily indirect and must remain so until direct measurement of protein folding becomes experimentally possible. Three main results have emerged. First, we studied the problem using simple HP models. It was found that sequences that folded more successfully to the native conformation *in vivo* (cotranslationally) than* in vitro* tended to have a higher level of hydrophobicity at the N-terminus than the C-terminus. Second, in a set of real proteins, a positive correlation between ALR (Average Logarithmic Ratio), a measure of the extent to which residues form contacts with previously extruded residues and considered a surrogate for cotranslational folding [16, 17], and the difference (N−C) in terminus hydrophobicity was shown. Third, cotranslational protein folding software (SAINT, Sequential Algorithm Initiated at the Nitrogen Terminus [17]) was used to fold real proteins from the amino to the carboxyl end and the quality of the prediction found to correlate positively with N−C difference in terminus hydrophobicity. These three findings are summarised in Figure 1.

## 2. Methods

Methods used in each of the three studies are now detailed.

### 2.1. Terminus Hydrophobicity and Cotranslational Folding in HP Lattice Models

Residues in the so-called “HP model” are of two types: hydrophobic (H) and polar (P). Residue positions are restricted to discrete locations on a lattice, here a square lattice [18]. The total energy is defined as negative the number of contacts between hydrophobic residues, where a contact is a pair of residues adjacent in space but not in sequence [1]. Such simple models have been shown to exhibit fundamental characteristics of protein folding such as two-state cooperativity and hydrophobic collapse [1]. In the current study, we used HP sequences of length 20 and evaluated their propensity to fold cotranslationally. The distribution of hydrophobic residues in sequences that were found to fold more successfully * in vivo* than * in vitro* was then analysed.

An exhaustive list of length 20 HP sequences with a unique ground (or native) state is available from the authors of [19] (24,900 sequences, making up 2.4% of the population). Each sequence was folded in two ways: from a fully extended state * in vitro* and in cotranslational fashion * in vivo* as described below. Two hundred runs were performed using each method. Each run consisted of 10,000 moves; if the native state was found within that time, the run was considered to be successful. The proportion of successful * in vitro* and * in vivo* runs was then calculated for each sequence.

Three types of move were used in the simulation: “pull moves” as described in [20], “drift moves” consisting of the rotation of a single bond by 90° (displacing all residues on one side of the bond by a distance equal to the lattice diagonal), and “null moves” which leave the current conformation unchanged. Move selection was based on the Boltzmann distribution. All possible conformations reachable from a current conformation, together with their energy level were determined. The next move was chosen according to a probability distribution defined in the following way. The probability of selecting a move with energy *E* is *K* times the probability of selecting a move with energy *E* + *C*, where *K* = *e* ^*C*/*T*^ and *T* is the temperature. A fixed temperature of 0.3 was used, since this results in a stable native structure for the majority of the sequences. Sequence stability was measured as the probability of returning to the same structure after four moves.

Cotranslational folding was performed as follows. Five N-terminus residues were initially extruded in a fully extended conformation. Twenty moves were performed, then the next residue extruded. After each extrusion, 20 + 7  (current  length − 5) additional moves were performed, giving a total of 1035 moves before full length was reached. The linear increase in the number of moves after each extrusion reflects the fact that in a real protein, all residues move simultaneously; allocating a number of moves proportional to the current length results in approximately the same amount of “movement” at each residue position. A further 8965 moves were then executed. As in [21], the ribosome surface was modelled as a half plane; previous residues were forbidden to move to the left of the most recently extruded residue. This restriction was lifted as soon as the peptide was fully grown and the final residue extruded. *In vitro* folding was straightforward; it began with a fully extended chain to which 10,000 moves were applied, so allotting *in vitro* and *in vivo* folding the same number of moves.

Following 200 such runs, the* in vitro* and* in vivo* success rate for each sequence was determined. Each sequence was then assigned to one of five groups: *C* (*in vivo* minus* in vitro* success rate at least 20%), *I* (*in vitro* minus* in vivo* success rate at least 20%), *G* (both good, each with a success rate higher than 95%), *B* (both bad, each with a success rate lower than 5%), or *N* (none of the above). The distribution of hydrophobic residues in the first four groups was then analysed. Groups *I* and *C* were based on a comparison of the two success rates, rather than on a single success rate, because sequences with a very high success rate tend to perform well no matter how they are folded. The sequences of interest are those that have a substantial advantage (or disadvantage) when folded cotranslationally compared to *in vitro*. We also calculated the mean N−C terminus difference in hydrophobicity of the HP sequences, where the N-terminus and C-terminus comprised the first four and last four residues respectively. Interest was in whether* in vivo* less *in vitro* success rate increases with this difference.

### 2.2. Terminus Hydrophobicity and ALR for Real Proteins

With HP models, we can observe and control their folding. We cannot even observe the folding of real proteins, so we must infer folding behaviour from other measures. One such surrogate, ALR (Average Logarithmic Ratio) [16], measures the degree of previous contacts of a sequence from each end and takes the logarithm of the ratio of previous contacts at the N-terminus to previous contacts at the C-terminus. A previous N-terminus contact occurs with a residue at position *j* ≥ 7 from the N-terminus when a residue at postion 1 to *j* − 6 is within 13 Å of residue *j*. (We ignore the five closest residues towards the N-terminus as contact candidates, because such contacts are generally due to closeness in sequence rather than the folding process.) Similarly, a previous C-terminus contact occurs with a residue at position *j* ≥ *n* − 6 (where *n* is the position of the C-terminus) when a residue at position *j* + 6 to *n* is within 13 Å of residue *j*. The actual number of previous contacts of residue *j* with residues towards the N-terminus is denoted by *A*_*j*_^N^, while *P*_*j*_^N^ = *j* − 6 represents the potential number of such contacts. The ratio of actual contacts to potential contacts for each residue, from the seventh onwards, is formed. These ratios are then compared to the corresponding ratios formed from the C-terminus.

To avoid zeros, residues are grouped until sums of actual contacts from both termini are nonzero. So, as the chain is parsed simultaneously from the nitrogen and carbon termini, if both *A*_*j*_^N^ and *A*_*j*_^C^ are greater than zero, a group is formed. Otherwise, parsing of the chain continues until the sum of all current *A*_*j*_^N^ values and the sum of all *A*_*j*_^C^ values are both greater than zero. We let *J*_*i*_ denote the number of residues in the *i*th group. Then, ALR is defined as the average of the logarithm of the resulting *A*_*j*_^N^ to *A*_*j*_^C^ ratios, over the *I* groups,

(1) $$\begin{array}{c} \text{ALR}=\frac{1}{I}\overset{I}{\underset{i=1}{\sum }}\log\;\left(\frac{\sum_{j=1}^{J_{i}}A_{j}^{\text{N}}}{\sum_{j=1}^{J_{i}}A_{j}^{\text{C}}}\right) \end{array}.$$

We can also measure the (N−C) difference in terminus hydrophobicity in real proteins. A nonredundant database of protein conformations (<30% sequence identity, resolution better than 3 Å, at least 100 residues, and no missing residues, downloaded on 6 February 2009) from the PISCES [22] server was used to create test sets. One set contained protein chains with ALR of zero or greater (likely to fold cotranslationally, a total of 45 proteins), while the second set contained protein chains with ALR less than zero (likely to fold noncotranslationally, a total of 45 proteins). There are several scales that measure hydrophobicity, but for our primary analysis, we used the Kyte-Doolittle scale [23]. This has values greater than zero for residues that are hydrophobic and values less than zero for residues that are hydrophilic. It is based on experimental data of the physicochemical properties of amino acid side chains and is useful for defining surface-exposed regions. To check that results are not dependent on this particular choice of scale, we ran the same analysis with other popularly employed hydrophobicity scales, the Janin [24], Cornette et al. [25], and Eisenberg et al. [26] scales. The first 10 residues in the protein sequence constituted the N-terminus and the last 10 the C-terminus. The mean hydrophobicity was calculated for the N-terminus and similarly, the mean calculated for the C-terminus. The difference between the means (N−C) was computed for each protein. A lowess curve was drawn through the plot of ALR against difference in mean terminus hydrophobicity to check for association. 

### 2.3. Terminus Hydrophobicity and SAINT for Real Proteins

Rather than use a surrogate measure, we chose to use the SAINT [17] cotranslational protein structure prediction software to more directly measure cotranslational folding success. As outlined in [17], this measure can be strengthened by using the “forward minus backward” difference in GDT_TS (Global Distance Test Total Score), the GDT_TS of the SAINT predicted conformation to the native conformation less the GDT_TS of the reverse SAINT predicted conformation to the native conformation. (GDT_TS is a convex combination of the proportions of the C*α* model atoms within 1, 2, 4, and 8 Å of the corresponding native atoms, following superpositions [27].) Reverse SAINT predicts the conformation as if the protein were extruded from the C rather than the N-terminus. This is analogous to use of a pairwise comparison in statistical design.

By way of background, in [17], a set of 1000 models was generated for each of 68 proteins with both SAINT and reverse SAINT. GDT_TS was employed to assess model quality. On average, SAINT produced models of higher mean GDT_TS for positive ALR proteins than it did for negative ALR proteins. SAINT also generally performed better than reverse SAINT. Data from this study was used in the current study.

Terminus regions for the 68 proteins in [17] were deemed to be the first 10 residues (for the N-terminus) and the last 10 residues (for the C-terminus). Hydrophobicity of terminus regions was assigned using the Kyte-Doolittle scale and the N-terminus mean and C-terminus mean calculated. The difference between mean GDT_TS of SAINT and mean GDT_TS of reverse SAINT (where the mean is calculated over 1000 models for each protein) was plotted against mean difference in terminus hydrophobicity for the set of sequences.

## 3. Results

The three main results are now separately described.

### 3.1. Terminus Hydrophobicity and Cotranslational Folding in HP Lattice Models

Of the 24,900 sequences with a unique global minimum, 716 had cotranslational success rate minus* in vitro* success rate of at least 20% (group *C*), 1012 had* in vitro* success rate minus cotranslational success rate of at least 20% (group *I*), 412 had a success rate above 95% both cotranslationally and* in vitro* (group *G*), and 794 had a success rate below 5% for both (group *B*). (A caution: while the role of cotranslational folding is increasingly being acknowledged, we should not infer that the proportion of real proteins favouring cotranslational folding is as found in this very limited model situation.)

**Figure 2:**
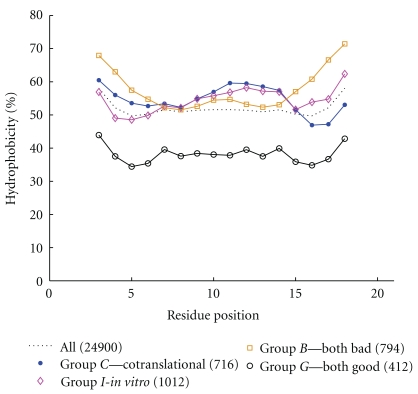
The (smoothed) percentage of hydrophobic residues at each position (1 is the N-terminus) for each class of sequence. Data is smoothed with a window size of five, centred on the given residue. The dotted line is for all HP sequences of length 20 with a unique global minimum. The other four lines correspond to groups *C*, *I*, *B*, and *G*, as described in the text. Of greatest interest here is the fact that hydrophobicity in the nitrogen terminus region for group *C* is higher than that in the carbon terminus region.

Figure 2 shows the (smoothed, with a window size of five) percentage of residues which are hydrophobic, for each residue position, from the start to the end of the sequence, for all sequences and then each of groups *C*, *I*, *B*, and *G*. We draw out three observations from this graphic. First, for all 24,900 sequences (the dotted line in Figure 2), termini are more hydrophobic than the central region, suggesting that this property is generally required for possession of a unique global minimum, a feature of stability. The symmetry of this line is due to the fact that both the forward and reverse versions of each sequence are represented. Second, good folders (group *G*) have the lowest overall hydrophobicity, and bad folders (group *B*) have the highest, with the difference most evident at the termini. An interpretation is that a protein with higher density of hydrophobic residues has an energy surface possessing more local minima, providing the potential to “trap” the folding protein before it reaches the native state. We also remark that bad folders *B* are less hydrophobic than cotranslational folders *C* in the central bulk but more hydrophobic at the termini. Collectively, these observations suggest that terminus hydrophobicity primarily aids stability of the native conformation but stands in the way of efficient folding. Third, hydrophobicity tends to decrease from the N to C-terminus for the good cotranslational folders *C* but increase for the good fully extended folders *I*. Though apparently a second-order effect, this observation is the one of greatest interest in this paper. Finally, note that it is the average (across a window size of five, so plus or minus two residues from each position) hydrophobicity across the sequence that is shown for each group in Figure 2. (We remark that use of a window size of three or seven does not markedly alter these conclusions.)

The third-mentioned result of Figure 2 triggered the investigation of this paper. The difference between the percentage of H residues in the first five residues of all 716 *C* sequences and the percentage of H residues in the final five residues is 7.4% (note that this is a difference between population percentages).

**Figure 3:**
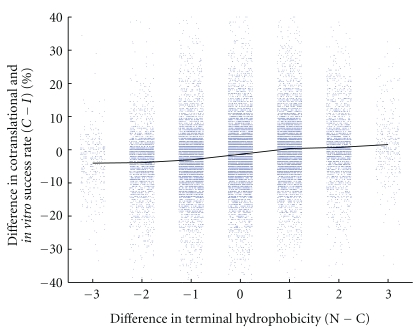
Difference in success rate (cotranslational minus * in vitro*) against difference in terminus hydrophobicity (N−C) for all 24,900 sequences of length 20. The horizontal axis shows the number of H residues in the first four positions of the sequence (the N-terminus region) minus the number of H residues in the last four positions (the C-terminus region). Data points have been jittered horizontally to clarify their distribution.

We proceeded to investigate whether cotranslational folding itself was associated with an N−C terminus difference in hydrophobicity. In Figure 3, we show the result: the cotranslational minus* in vitro* success rate tends to increase as the difference between N and C-terminus hydrophobicity increases.

### 3.2. Terminus Hydrophobicity and ALR for Real Proteins

**Figure 4:**
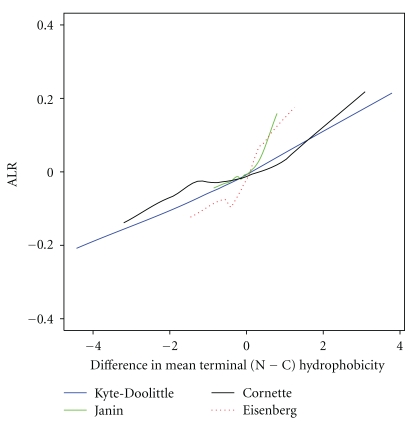
ALR (dominance of previous contacts) for 90 protein sequences from the PISCES set against (N−C) difference in mean terminus hydrophobicity. The N-terminus and C-terminus of these proteins are the first and last ten residues in their sequences, respectively. Hydrophobicity was assigned using the Kyte-Doolittle, Janin, Cornette, and Eisenberg scales. Note that ALR increases as the difference in mean terminal hydrophobicity increases, for all four scales.

Here, we investigate the relationship between the difference between N-terminus and C-terminus hydrophobicity and ALR [16], the dominance of previous contacts, in a set of 90 real proteins. High ALR ratios (>0) indicate potential cotranslationality. We find that ALR increases with the difference in mean terminus hydrophobicity, using any of the four hydrophobicity scales, as shown in Figure 4.

The causal relationship amongst four variables (N−C hydrophobicity difference, extent of cotranslational folding, extent of nitrogen terminus burial and ALR) underlies Figure 4. In the absence of cotranslational folding, it is plausible that extent of nitrogen terminus burial is driving both N−C hydrophobicity difference and ALR. The hypothesis adopted in this paper, and supported by the other findings, is rather that extent of N−C hydrophobicity difference drives extent of cotranslational folding which in turn buries the nitrogen terminus, so yielding a higher ALR.

### 3.3. Terminus Hydrophobicity and SAINT for Real Proteins

**Figure 5:**
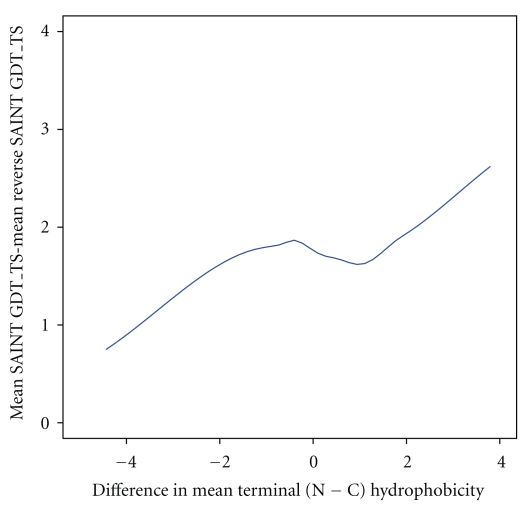
Showing that as the difference in mean terminus hydrophobicity increases, the difference between mean SAINT GDT_TS and reverse SAINT GDT_TS also increases. The curve shown is a lowess plot through the data based on 68 protein chains taken from [17]. Terminals comprised the first 10 (N-terminus) and last 10 (C-terminus) residues in the sequence. Mean hydrophobicity for each terminus was calculated using the Kyte-Doolittle scale [23]. Each protein was folded 1000 times using SAINT and reverse SAINT.

The extent of cotranslational folding is magnified by subtracting the performance (measured by GDT_TS) of reverse SAINT from that of SAINT. This cotranslationality measure is plotted against the difference in mean terminus hydrophobicity between the nitrogen and carbon termini (N−C) for 68 real proteins in Figure 5. Proteins with more hydrophobic N-terminus than C-terminus show greater evidence of successful cotranslational folding. 

## 4. Discussion

This section discusses certain results that consolidate those in the previous section, first for HP models, then for real proteins, concluding with some general remarks.

### 4.1. HP Models

**Figure 6:**
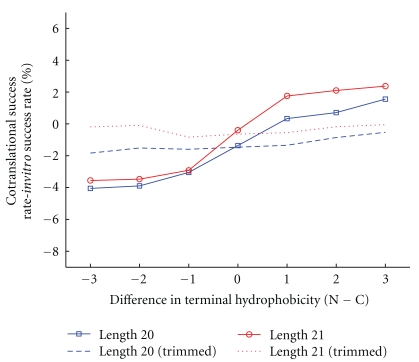
Difference between cotranslational and* in vitro* success rate against difference in terminus hydrophobicity (N−C) for HP lattice models. The blue lines (squares and dashes) are for all 24,900 sequences of length 20; the dashed line is for “trimmed” sequences, where the first and last four residues of each sequence are removed. The red lines (circles and dots) are for all 52,183 sequences of length 21; the red dotted line is for trimmed 21-mer sequences. Note that the positive relationship continues for 21-mers, but that it is largely lost when trimming occurs.

We first extended the study to the set of 52,183 21-mer HP sequences with a unique global minimum; a similar positive relationship to that for 20-mer sequences was found between cotranslational success and difference in terminus hydrophobicity, as shown in Figure 6. Further, to determine whether this aspect of protein folding is only an immediate end effect, we trimmed four residues from both ends of all sequences. This very considerably reduced the effect for 20-mers and totally removed it for 21-mers (the dashed and dotted line in Figure 6, respectively). 

**Figure 7:**
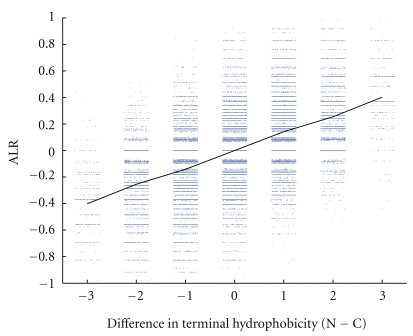
ALR against difference in terminus (N−C) hydrophobicity for HP lattice models of length 20. Data points have been jittered horizontally. A positive relationship is evident, as was found earlier for real proteins.

We found (Figure 4) that for real proteins, ALR was positively related to difference in terminus hydrophobicity. A version of ALR was developed for HP models and a similar relationship to difference in terminus hydrophobicity was found, as shown in Figure 7. The quantity ALR for HP models is defined as log (*NC*/*CC*), where *NC* is the number of residues with at least one previous N-terminus contact, and *CC* is the number of residues with at least one previous C-terminus contact. A contact is defined as adjacency in space but not in sequence. If there is a “turn” at a residue (that is, the previous residue, this residue, and the next residue do not lie in a straight line), then diagonally adjacent residues are considered to be in contact.

### 4.2. Real Proteins

First, we examine the response of ALR to terminus hydrophobicity difference as the terminus extends, as well as the effect of end trimming. Second, we examine cotranslationality success as the terminus extends and as the ends are trimmed.

Terminus regions were 10 residues long in our preliminary analysis. Terminus regions of 20 and 30 residues are considered here also, in order to invesigate whether the relationship between ALR and terminus hydrophobicity persists (shown first in Figure 4) when termini are lengthened. In addition, in order to determine the importance of ends, 10% of the length of each protein was trimmed from each end, the remainder divided into quartiles, and then the first and fourth quartiles deemed the N and C termini, respectively.

**Figure 8:**
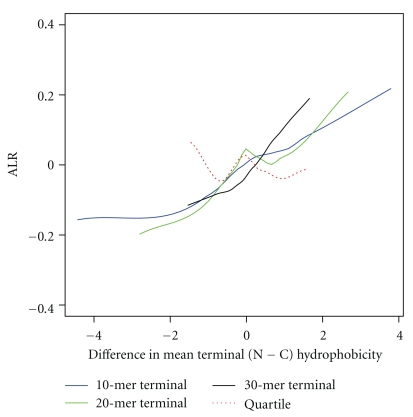
ALR against difference in terminus hydrophobicity (N−C) is shown for real proteins (90 in total). The solid lines represent proteins whose N and C-termini were defined as being the first and last 10 (blue), 20 (green), and 30 (black) residues in their sequence. The dashed red line indicates that terminus regions were defined as the first (N) and fourth (C) quartiles of a sequence after the trimming of 10% from their lengths at each end. Note that the positive relationship remains for all terminus lengths, but that is lost when the ends are removed.

A positive relationship between ALR and terminus hydrophobicity difference for termini of all studied lengths is evident, but not when end residues are removed, as shown in Figure 8. 

**Figure 9:**
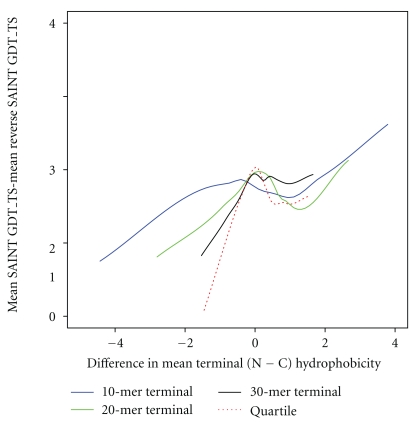
The difference in mean SAINT GDT_TS and mean reverse SAINT GDT_TS against difference in terminus hydrophobicity (N−C) is shown for real proteins (90 in total). The solid lines represent proteins whose N and C termini were defined as being the first and last 10 (blue), 20 (green), and 30 (black) residues in their sequence. The dashed red line is for proteins whose terminus regions were defined as the first (N) and fourth (C) quartiles of a sequence after the trimming of 10% from their lengths at each end.

For the same terminus lengthening and end removal situations, the association between the difference in SAINT and reverse SAINT mean GDT_TS and the difference in mean terminus hydrophobicity was investigated, extending the earlier result of Figure 5. The relationship is still present, but less distinctly, as illustrated in Figure 9. 

### 4.3. General Discussion

**Figure 10:**
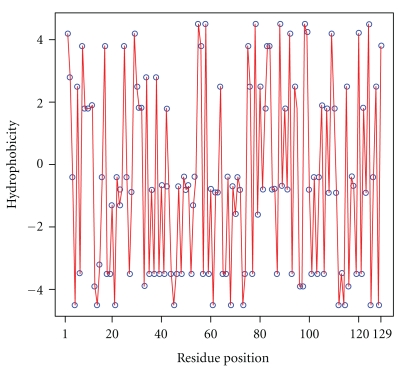
The hydrophobicity profile of the 129-mer hen egg-white lysozyme discussed in [2].

Given the seminal nature of the work of Phillips, it is of interest to determine whether there is a difference in mean terminus hydrophobicity in the hen egg-white lysozyme. The Kyte-Doolittle hydrophobicity profile is shown in Figure 10. With terminus regions 10 residues in length, there is a 0.16 difference in mean terminus hydrophobicity, with the N-terminus being more hydrophobic than the C-terminus. This supports the early statement of Phillips that this structure does appear to show evidence of cotranslational folding. 

Finally, we comment on the work of White and Jacobs [3]. A key finding was that at least 60% of proteins studied had a random hydrophobicity profile. We have demonstrated here that if we separate out (and we have done this in a variety of ways) those that are considered to fold cotranslationally, then a hydrophobicity pattern is seen. These findings are not contradictory. 

## 5. Conclusions

Capturing sequences which fold cotranslationally is a challenge. In this paper, we have done this in three ways, through folding HP models and noting sequences which successfully fold cotranslationally, through the surrogate measure of ALR for real proteins and through the cotranslational folding software SAINT for real proteins. In each case, sequences which fold well cotranslationally are associated with a fore to aft decline in hydrophobicity. Once seen, this is not surprising, since an initial hydrophobic segment which prefers to be in the interior of the conformation, followed by a final less hydrophobic segment that prefers to be on the exterior of the conformation, can be expected to fold efficiently in a sequential manner.

In conclusion, we found consistent evidence in three independent ways, via HP lattice models, ALR and the SAINT software, that greater hydrophobicity at the N-terminus than the C-terminus can drive cotranslational folding. A concern about incorporation of cotranslation into protein fold prediction is that of determining sequences for which it is relevant. The terminus hydrophobicity drop described here is a further measure which could be used to indicate when such an approach to fold prediction may be appropriate.
